# Trends in Continuous Deep Sedation until Death between 2007 and 2013: A Repeated Nationwide Survey

**DOI:** 10.1371/journal.pone.0158188

**Published:** 2016-06-23

**Authors:** Lenzo Robijn, Joachim Cohen, Judith Rietjens, Luc Deliens, Kenneth Chambaere

**Affiliations:** 1 End-of-life Care Research Group, Department of Family Medicine and Chronic Care, Vrije Universiteit Brussel (VUB) & Ghent University, Brussels, Belgium; 2 Department of Medical Oncology, Ghent University Hospital, Ghent, Belgium; Azienda Ospedaliero-Universitaria Careggi, ITALY

## Abstract

**Background:**

Continuous deep sedation until death is a highly debated medical practice, particularly regarding its potential to hasten death and its proper use in end-of-life care. A thorough analysis of important trends in this practice is needed to identify potentially problematic developments. This study aims to examine trends in the prevalence and practice characteristics of continuous deep sedation until death in Flanders, Belgium between 2007 and 2013, and to study variation on physicians’ degree of palliative training.

**Methods:**

Population-based death certificate study in 2007 and 2013 in Flanders, Belgium. Reporting physicians received questionnaires about medical practices preceding the patient’s death. Patient characteristics, clinical characteristics (drugs used, duration, artificial nutrition/hydration, intention and consent), and palliative care training of attending physician were recorded. We posed the following question regarding continuous deep sedation: ‘Was the patient continuously and deeply sedated or kept in a coma until death by the use of one or more drugs’.

**Results:**

After the initial rise of continuous deep sedation to 14.5% in 2007 (95%CI 13.1%-15.9%), its use decreased to 12.0% in 2013 (95%CI 10.9%-13.2%). Compared with 2007, in 2013 opioids were less often used as sole drug and the decision to use continuous deep sedation was more often preceded by patient request. Compared to non-experts, palliative care experts more often used benzodiazepines and less often opioids, withheld artificial nutrition/hydration more often and performed sedation more often after a request from or with the consent of the patient or family.

**Conclusion:**

Worldwide, this study is the first to show a decrease in the prevalence of continuous deep sedation. Despite positive changes in performance and decision-making towards more compliance with due care requirements, there is still room for improvement in the use of recommended drugs and in the involvement of patients and relatives in the decision-making process.

## Introduction

Physicians caring for patients with an advanced disease are often confronted with important but complex end-of-life decisions that affect the patient’s manner of dying [1]. When terminally ill patients experience unbearable symptoms that cannot be alleviated by conventional treatments, administering drugs to induce unconsciousness can be an option of last resort to relieve suffering [2,3]. Large-scale population-based surveys monitoring end-of-life practices on a nationwide scale have so far consistently shown an increased use of continuous deep sedation until death. In Flanders, the overall prevalence of this practice increased considerably between 2001 and 2007, rising from 8.2% to 14.5% of all deaths [4,5]. In the Netherlands, studies found that the prevalence of continuous deep sedation increased from 8.2% in 2005 to 12.3% of all deaths in 2010 [1,6,7].

Continuous deep sedation until death remains a highly debated medical practice, particularly regarding its potential to hasten death and its proper use in end-of-life care [8–10]. In light of the clinical and ethical challenges associated with the practice, several guidelines and recommendations have been developed around the world. In Flanders the Federation for Palliative Care implemented a guideline in 2010 [11], describing conditions under which sedation at the end of life should be performed [12–14]. Like guidelines in many other countries, it recommends that continuous deep sedation until death should only be performed close to death for unbearable and refractory symptoms without intent to hasten death [15,16]. Benzodiazepines, titrated proportionally to alleviate the symptoms, are the drug of first choice and the administration of artificial nutrition or hydration is not encouraged unless the benefits outweigh the harm [4,12].

Empirical studies indicate that there is considerable variation regarding this medical practice and that physicians are not always well acquainted with the conditions under which continuous deep sedation until death should be performed [17]. The effectiveness of guidelines, being non-committal and non-mandatory, in changing physicians’ attitudes, knowledge and practices have been called into question [18]. In the Netherlands, where the Royal Dutch Medical Association issued a clinical guideline in 2005 and a revised guideline in 2009 [19], studies have suggested that guidelines can certainly lead to considerable practice improvements in accordance with guideline requirements [20]. Some of the results even suggest better compliance with the guidelines when physicians had more palliative care expertise [21]. This might also be the case in Belgium after the introduction of the Flemish guideline by the Federation for Palliative Care in 2010. This study describes recent developments in the prevalence and characteristics of continuous deep sedation until death in Belgium between 2007 and 2013 and studies variation in performance and decision-making depending on the degree of palliative care training of the physician.

## Methods

### Study design

We conducted a population-based death certificate survey identical to surveys in 1998, 2001 and 2007, based on a representative sample of deaths in Flanders, Belgium. This region has approximately six million inhabitants and 60.000 deaths annually [4,22]. To limit the time between the certification of death and the inclusion in the study, a stratified random sample of deaths in 2013 was drawn weekly from the Flemish Agency for Care and Health, the central administration authority for processing death certificates. From our previous studies [5,23,24] we know that end-of-life decisions occur more frequently among patients with a certain cause of death. We therefore adopted disproportionate sampling of deaths to include more patients with a cause of death known to have a higher likelihood of one or more end-of-life decisions. All deaths from January 1^st^ until June 30^th^ 2013 of Belgian residents aged one year or older were assigned to one of three strata, based on underlying cause of death as indicated on the death certificate and the estimated corresponding likelihood of an end-of-life decision. Sampling fractions for each stratum increased with this likelihood [23]. In the first stratum, all deaths for which euthanasia was mentioned on the death certificate were sampled. In the second stratum, one third of all cancer deaths were sampled. In the third stratum, one in six deaths resulting from any other cause was sampled. This resulted in a sample of 6.871 deaths, about 21% of all deaths in the studied period.

Within two months of the death, the certifying physician received a four-page questionnaire with an introductory letter containing patient identifiers. The physician was requested to complete the questionnaire by consulting the patient’s medical file. If the certifying physician was not the treating physician, the questionnaire was passed on to the treating physician. One physician could receive participation requests for up to five decedents, with at most three reminders per death; every sixth case was excluded and another death was sampled from the same stratum and the same place of death. To guarantee absolute anonymity for participating physicians, a lawyer served as an intermediary between responding physicians, researchers and the Flemish Agency for Care and Health, ensuring that completed questionnaires could never be linked to a particular patient or physician. Patients were deceased, and consent was not required. Physicians’ participation was regarded as implicit consent, which was noted in the accompanying letter introducing the study. After data collection a one-page questionnaire was mailed to all non-responding physicians inquiring about reasons for not participating. The mailing and anonymity procedures were approved by the Ethical Review Board of the University Hospital of the Vrije Universiteit Brussel, the Belgian National Disciplinary Board of Physicians and the Belgian Privacy Commission.

### Questionnaire

The repeatedly validated questionnaire on end-of-life decision-making first asked whether death had been sudden and unexpected. The rest of the questionnaire was to be completed only if death had not been sudden and unexpected. The following question, identical to that used in 2001 [22,25] and 2007 [23], was posed regarding continuous deep sedation: *Was the patient continuously and deeply sedated or kept in a coma until death by the use of one or more drugs*?. We used a descriptive definition of the practice (continuous deep sedation until death) rather than a term (palliative or terminal sedation) to avoid interpretation differences among respondents. The physician’s degree of palliative training is coded if he/she reported that they (1) had not had palliative care training; (2) had only had some palliative care training in the basic curriculum; (3) had followed continued palliative care training or (4) worked as part of a palliative care team (palliative care experts). Demographic and clinical patient data were obtained from the death certificate data and linked anonymously after data collection.

### Statistical analysis

The response sample was corrected for disproportionate stratification by weighting each stratum to make the proportion in the response sample identical to the proportion in all deaths and adjusted to be representative of all deaths in the first half of 2013 in terms of age, sex, marital status, province of death, cause of death and place of death (adjustments needed for province and place of death). After this weighting procedure there were no significant differences between response sample and all deaths in any of these variables. Final weights varied between 0.11 and 1.90. This procedure was also used in previous survey years. Bivariate cross-tabulations and multivariable logistic regression models were calculated to compare prevalence and characteristics of continuous deep sedation between 2007 and 2013. Multivariable models incorporated the most important confounders: sex, age, cause of death and place of death. All statistical analyses were calculated with complex samples functions in SPSS version 22.0.

## Results

Of the 6.871 deaths sampled, questionnaires were returned for 3.751 cases. From the non-response analysis we found that response was impossible for 683 deaths (e.g. because the physician did not have access to the patient’s medical file or the patient could not be identified). These cases were removed from the sample. Response rate was therefore 60.6% (3.751/6.188 eligible cases) compared with 58.4% in 2007. Analysis of non-response questionnaires revealed lack of time as the most quoted reason for non-participation. Between 2007 and 2013 there was an increased proportion of decedents aged 80+ from 50.0% to 57.1% and deaths in nursing homes rose from 22.6% in 2007 to 26.9% in 2013 (data not shown). Cancer consistently accounted for around one in four deaths.

The overall prevalence of continuous deep sedation decreased significantly from 14.5% (95%Cl 13.1–15.9) to 12.0% (95%Cl 10.9–13.2, p = 0.007) (Table 1). The decreasing trend is visible in nearly all patient groups, but is statistically significant only in women (from 15.4% [95%Cl 13.5–17.6] in 2007 to 11.8% [95%Cl 10.3–13.6] in 2013), in decedents of 80 years or older (from 11.1% [95%Cl 9.4–13.0] to 8.9% [95%Cl 7.6–10.3]), in persons with primary diagnoses other than cancer (12.9%[95%Cl 11.2–14.8] to 10.4% [95%Cl 7.6–10.3]), in decedents with a high school or college/university degree (from 18.5% [95%Cl 15.3–22.2] to 13.0% [95%Cl 10.6–15.8]), among widowed decedents (11.8% [95%Cl 9.9–14.0] to 8.4% [7.0–10.1]) and among those living in care homes (9.4% [95%Cl 7.4–11.8] to 6.6% [95%Cl 5.2–8.4]). The decrease remained significant after simultaneously controlling for relevant confounding factors for the total prevalence of continuous sedation (p = 0.037), among women (p = 0.017), in patients with a high school or college/university degree (p = 0.019), among widow(er)s (p = 0.022) and those dying in care homes (p = 0.035).

**Table 1:** Prevalence of continuous deep sedation until death (CDS) and baseline characteristics of patients receiving CDS between 2007 and 2013^a^,^b^.

	*Number of cases*		*Weighted percentages*		*Biv*. *P-value*
	2007	2013	2007	2013	
	N = 3623	N = 3751	% (95%Cl)	% (95%Cl)	
**Total CDS**	561	438	**14.5 (13.1–15.9)**	**12.0 (10.9–13.2)**	**0.007**
**Sex**					
Male	1875	1920	13.5 (11.8–15.6)	12.2 (10.6–13,9)	0.275
Female	1748	1826	**15.4 (13.5–17.6)**	**11.8 (10.3–13.6)**	**0.006**
**Age (in years)**					
1–64	741	632	19.3 (16.1–23.0)	16.5 (13.5–20.0)	0.245
65–79	1267	1100	17.1 (9.5–14.0)	16.0 (13.7–18.6)	0.540
80+	1615	2014	**11.1 (9.4–13.0)**	**8.9 (7.6–10.3)**	**0.050**
**Cause of death**					
Cancer	2018	1470	18.4 (16.6–20.3)	16.6 (14.7–18.8)	0.222
Non-cancer	1605	2258	**12.9 (11.2–14.8)**	**10.4 (9.1–11.9)**	**0.032**
**Education**					
Primary school	1196	923	13.3 (11,2–15,8)	10.2 (8.2–12.6)	0.054
High school (not graduated)	692	639	13.7 (10.8–17.3)	12.5 (10.0–15.7)	0.598
High school/college	726	778	**18.5 (15.3–22.2)**	**13.0 (10.6–15.8)**	**0.010**
**Marital status**					
Unmarried	357	372	12.0 (8.6–16.5)	8.7 (6.1–12.3)	0.192
Married	1798	1618	17.5 (15.4–19.9)	15.9 (14.0–18.0)	0.296
Widow(er)	1252	1445	**11.8 (9.9–14.0)**	**8.4 (7.0–10.1)**	**0.008**
Divorced	214	305	13.0 (8.6–19.0)	14.7 (10.8–19.6)	0.629
**Setting** ^c^					
At home	1265	1133	9.8 (8.3–11.6)	8.7 (7.2–10.4)	0.336
Hospital ^d^	1382	1447	19.5 (17.2–22.0)	17.0 (15.0–19.1)	0.120
Care Home	850	1038	**9.4 (7.4–11.8)**	**6.6 (5.2–8.4)**	**0.037**

^a^ Figures are weighted percentages of all deaths and 95% confidence intervals. Figures in bold denote statistically significant differences between 2007 and 2013.

^b^ After controlling for the most important confounders (age, sex, cause of death and place of death) differences in the following groups between 2007 and 2013 remained significant: total CDS, female, high school/college, Widow(er) and care home. The direction of bivariate associations did not change in multivariate analysis.

^c^ Other place of death not included in table: 13 cases in 2007 and 12 cases in 2013.

^d^ In 2013, we could distinguish different departments within the hospitals. In 2013, continuous sedation until death within the hospital was more often used in an intensive care unit (50.5%, 95%CI 43.8–57.6) than in a palliative care unit (23.9%, 95%CI 16.7–32.9) (p<0.001).

Benzodiazepines and opioids were the most frequently used drug combination in 2007 and 2013, and opioids were less often used in 2013 as sole drug (Table 2). Compared to 2007, sedation in 2013 was more often performed with propofol (23.1% vs 11%). The duration of sedation was relatively shorter in 2013 compared with 2007, with a higher proportion of continuous deep sedations lasting less than 24 hours (35.8% vs 24.4%). Though artificial nutrition or hydration was less often administered until death in 2013 (38.3% vs 42.5% in 2007), multivariable analysis showed this was due the increased proportion of decedents aged 80 and over, for whom artificial nutrition or hydration is less likely (not in table). Sedation was more often performed after a request from the patient in 2013 (15.3%) than in 2007 (9.7%). The lacking of patient or family consent mainly occurred in the hospital setting (89.4% vs 93.4% in 2007) and in persons with primary diagnoses other than cancer (78.6% vs 81.5% in 2007) such as cardiovascular diseases (35.1% vs 37.6% in 2007) (not in table). No significant differences were found regarding the intention of hastening death between 2007 and 2013. In 2013, the life-shortening effect of sedation was explicitly intended or co-intended in 17.9% of cases.

**Table 2:** Characteristics of performing continuous deep sedation until death in 2007 and 2013^a^ ^b^.

	N		Total CDS		Chi^2^ P-Value ^c^^,^^d^
	2007	2013	2007	2013	
	N = 561	N = 438	%	%	
**Drugs administered**					**<0.001**
Only benzodiazepines	72	52	11.2	10.5	
Benzodiazepines and opioids (+other drugs)	239	213	42.4	46.2	
Propofol (+benzodiazepines/opioids/other drugs)	32	73	11.0	23.1	
Only opioids	167	79	30.7	16.7	
Other combinations	24	16	4.7	3.5	
**Duration of sedation**					**<0.001**
0–24 hours	125	153	24.4	35.8	
1–7 days	321	247	61.7	54.5	
1–2 weeks	58	21	11.2	6.0	
More than 2 weeks	12	12	2.7	3.7	
**Artificial nutrition and hydration**					**0.038**
Administered until death	159	129	42.5	38.3	
Withdrawn during sedation	43	49	9.4	12.5	
withheld	347	258	48.1	49.2	
**Request or consent**					0.095
Request by patient	71	83	9.7	15.3	
No request, but consent of patient	135	100	20.3	19.5	
No request or consent of patient, but request by family	78	60	11.8	13.8	
No request or consent of patient, but consent of family	186	131	38.4	35.2	
No request or consent of patient or family	74	54	19.8	16.2	
**Intention of hastening death**					0.329
No intention	124	99	32.4	29.2	
Taking into account possible hastening of death	280	236	51.2	52.9	
Co-intention	77	64	12.9	15.2	
Explicit intention	18	14	1.1	2.7	

^a^ Figures are weighted column percentages. Percentages may not always amount to 100% because of rounding. Figures in bold denote statistically significant differences between 2007 and 2013.

^b^ Missing cases: drugs administered (26 in 2007 and 5 in 2013), duration of sedation (45 in 2007 and 5 in 2013), artificial nutrition and hydration (12 in 2007 and 2 in 2013), request or consent (17 in 2007 and 10 in 2013) and intention of hastening death (62 in 2007 and 25 in 2013).

^c^ After controlling for the most important confounders (age and place of death), differences in ‘artificial nutrition and hydration’ can be attributed to the increased proportion of decedents aged 80 and over.

^d^ P-values were calculated with Fisher’s exact test (in StatXact version 6).

The performance and decision-making characteristics of continuous deep sedation until death in 2013 differed according to the degree of the physician’s palliative care expertise (Table 3). The use of benzodiazepines increased with palliative care expertise, whereas the use of opioids and propofol decreased (p<0.001). Those with training or expertise also withheld artificial nutrition or hydration more often (p<0.001) and sedation by palliative care experts was more often preceded by a request of the patient (p = 0.025) and less often without any request or consent of the patient or his or her family (p = 0.001). All significant bivariate results were also found significant after controlling for the most important confounders: sex, age, cause of death and place of death. The direction of bivariate associations did not change in multivariate analysis.

**Table 3:** Performance and decision-making characteristics of continuous deep sedation until death in 2013 according to the degree of physicians’ palliative care (PC) expertise^a^,^b^,^c^.

	No PC training	PC training in the basic curriculum	Continuing PC training courses	Specialist	Biv. P-Value ^d^
	N = 126	N = 109	N = 138	N = 63	
**Drugs administered**					**<0.001**
Only benzodiazepines	7.8	4.2	14	22.0	
Benzodiazepines and opioids (+other drugs)	32.9	52.5	52.1	55.0	
Propofol (+benzodiazepines/opioids/other drugs)	36.2	30.1	11.7	0	
Only opioids	19.0	12.8	19.4	13.2	
Other combinations	4.1	0.4	2.8	9.8	
**Duration of sedation**					0.524
0–24 hours	33.3	38.4	36.5	35.9	
1–7 days	51.6	55.5	54.4	61.4	
1–2 weeks	9.6	3.4	6.1	2.8	
More than 2 weeks	5.6	2.7	2.9	0	
**Artificial nutrition and hydration**					**<0.001**
Administered until death	48.6	49.6	25.7	17.1	
Withdrawn during sedation	8.8	21.0	12.4	5.0	
withheld	42.6	29.4	61.9	77.9	
**Request or consent**					**0.009**
Request by patient	8.2	17.5	17.2	23.7	
No request, but consent of patient	21.1	15.2	21.1	21.7	
No request or consent of patient, but request by family	11.5	12.4	16.9	16.1	
No request or consent of patient, but consent of family	34.8	34.7	38.8	31.1	
No request or consent of patient or family	24.5	20.1	6.1	7.3	
**Intention of hastening death**					0.107
No intention	36.5	33.3	24.0	13.3	
Taking into account possible hastening of death	43.0	51.0	59.9	65.9	
Co-intention	17.2	13.2	13.7	18.4	
Explicit intention	3.3	2.4	2.4	2.4	

^a^ Figures are weighted column percentages. Percentages may not always amount to 100% because of rounding. Figures in bold denote statistically significant differences according to the degree of physicians’ palliative care expertise.

^b^ Missing cases: drugs administered (5), duration of sedation (5), artificial nutrition and hydration (2), request or consent (10) and intention of hastening death (25).

^c^ All significant bivariate results were also found significant after controlling for the most important confounders: sex, age, cause of death and place of death. The direction of bivariate associations did not change in multivariate analysis.

^d^ P-values were calculated with Fisher’s exact test (in StatXact version 6).

## Discussion

Our robust population-based study found that after the initial rise of continuous deep sedation until death between 2001 and 2007 from 8.2% to 14.5%, its use decreased to 12.0% in 2013. The decrease particularly occurred in women, widowed people, those dying in nursing homes and the more highly educated. In 2013, compared with 2007 opioids were less often used as sole drug and the decision to use continuous deep sedation was more often preceded by an explicit patient request. Compared to non-experts, palliative care experts more often used benzodiazepines and less often opioids, withheld artificial nutrition or hydration more often and more often performed sedation after a request or with the consent of the patient or family.

So far, large-scale population-based surveys estimating the prevalence or development of continuous deep sedation until death have consistently found an increase in its use [1,4,5,22,26,27]. This study is the first to show a decrease in the use of continuous deep sedation, with the prevalence dropping from 14.5% in 2007 to 12.0% in 2013. This decrease could be attributable to Flemish physicians’ and other health care workers’ increased training and experience in palliative care and in controlling distressing symptoms without the need to use continuous deep sedation as an option of last resort. The decrease of continuous sedation may also be related to the specific Belgian context of end-of-life decision-making where euthanasia—defined as medical administration of life-ending drugs at the patient’s explicit request—is legal under a number of conditions [28]. A recent Belgian study found increasing numbers of euthanasia requests and granting rates between 2007 and 2013 [5]. This increase mainly took place in the same subgroups in which the present study found the use of continuous deep sedation to have substantially decreased during the same period [29]. There is evidence that in Flemish clinical practice euthanasia and continuous deep sedation are often discussed as alternative options, the choice between them depending on the preferences of patients and others involved [16,30,31]. It thus seems that the option of euthanasia is now chosen more often, due possibly to an increasing acceptance of euthanasia by patients, as well as by physicians and care institutions who in the past may more often have converted euthanasia requests into continuous deep sedation [32,33]. Other possible explanations for the decrease in continuous deep sedation are that ongoing ethical and clinical insights may have led to the view that the practice of continuous sedation is not ‘normal’ end-of-life treatment holding back some physicians from using it [13,16,34], or that increased attention to advance care planning, when patients are still capable of participating in end-of-life decisions, has reduced instances in which continuous sedation is performed as a crisis intervention in the absence of clear preferences or directives [35,36].

Our study found a number of striking changes in the performance of and decision-making preceding continuous deep sedation: in 2013, more sedations were carried out using a combination of benzodiazepines and opioids, with opioids less frequently used as sole drug than in 2007 and sedation was more often performed after a patient’s request, even though patient or family consent was still often lacking. In general, our study observed a number of developments in the practice of continuous deep sedation between 2007 and 2013 which are favourable in light of the recommendations described in the existing guidelines, including the 2010 Flemish guideline. This would corroborate research from the Netherlands showing that the practices of care providers had been positively influenced by the introduction of the Dutch guideline, first published in 2005, though the Dutch practice seems to fit more closely with the recommendations of the Dutch guideline than does the Flemish practice with the Flemish guideline [19,20,37]. There is still no insight into whether and to what extent guidelines, being non-mandatory, are applied in Flanders, Belgium. The fact that the Flemish guideline is issued by the Federation responsible for palliative care, rather than by a medical or health care association, can be expected to limit their spread. Our study suggests that there still is room for further improvement, particularly in the use of recommended drugs, seeking consent and not intending to hasten death. This raises the question whether guidelines alone can ensure sound practice. Too much emphasis on guidelines may encourage routinisation and could obscure the vital importance of case-by-case-based decision-making [38]. Following the proposed safeguards of Quill et al [39] for ethically complex practices such as continuous deep sedation (i.e. obtaining informed consent, ensuring diagnostic and prognostic clarity, obtaining an independent second opinion and documenting and reviewing the processes to ensure accountability) some are therefore calling for the mandatory consultation of palliative care experts or even mandatory reporting of continuous deep sedation as is the case for regulated euthanasia in Belgium [34,40,41]. However, in this study, palliative care training was associated with end-of-life sedation practices more congruent with recommendations. Therefore, a feasible alternative to mandatory consultation or reporting could be to encourage and enhance physician training in palliative care. Dutch research has found that the choice of recommended drugs for continuous deep sedation until death was associated with the use of guidelines and with the care team including, or consulting with a palliative care expert [1,21]. This suggests that palliative care training may thus improve a physician’s skills in performing end-of-life sedation as well as encourage them to adopt a multidisciplinary approach and consult end-of-life experts for this practice.

Although our study uses a robust population-based sampling method, a number of study limitations have to be taken into account. While high response rates were achieved, we cannot exclude some degree of non-response bias. However, analysis of non-response questionnaires revealed lack of time as the most quoted reason for non-participation. Our study only provides information from the physician’s perspective, and does not permit in-depth case analysis. Recall bias may also have influenced results, although attempts were made to limit this by ensuring that the physician received the questionnaire no later than eight weeks after their patient’s death. Sensitivity of survey topics may introduce untruthful or socially desirable reporting, but this is unlikely in our study given the explicit guarantee of anonymity and the fact that physicians were well acquainted with the survey. To minimize possible differences in the perception of sedation among the respondents we provided them with a descriptive definition of the practice (continuous deep sedation until death). Most other studies use terms such as palliative or terminal sedation, which can have various connotations. Furthermore, it is not known when the palliative care training reported by the respondents took place, nor the extent and content of the training.

## Conclusions

The decreased use of continuous deep sedation until death in almost all patient groups may suggest the development of a more critical approach and a more cautious attitude towards the practice among Flemish physicians. The specific context of legal euthanasia in Belgium may also play a role, and more research into the influence of different legal and cultural contexts on performance of continuous deep sedation until death is recommended. Despite positive changes in performance and decision-making towards more compliance with due care requirements, there is still room for improvement. Future studies should focus on whether quality improvement initiatives like mandatory consultation and basic palliative care training would improve the practice.
